# All-Electronic Emitter-Detector Pairs for 250 GHz in Silicon

**DOI:** 10.3390/s21175795

**Published:** 2021-08-28

**Authors:** Kęstutis Ikamas, Dmytro B. But, Albert Cesiul, Cezary Kołaciński, Tautvydas Lisauskas, Wojciech Knap, Alvydas Lisauskas

**Affiliations:** 1 Institute of Applied Electrodynamics and Telecommunications, Vilnius University, LT-10257 Vilnius, Lithuania; albert.cesiul@ff.vu.lt; 2 Research Group on Logistics and Defense Technology Management, General Jonas Žemaitis Military Academy of Lithuania, LT-10322 Vilnius, Lithuania; 3 CENTERA Laboratories, Institute of High Pressure Physics PAS, 01-142 Warsaw, Poland; dbut@mail.unipress.waw.pl (D.B.B.); ckolacinski@mail.unipress.waw.pl (C.K.); knap.wojciech@gmail.com (W.K.); 4 CEZAMAT, Warsaw Technical University, 02-822 Warsaw, Poland; 5 MB “Terahertz Technologies”, LT-01116 Vilnius, Lithuania; tautvydas.lisauskas@gmail.com

**Keywords:** opto-pair, CMOS, voltage-controlled oscillator, THz detection, THz imaging, field-effect transistor

## Abstract

The spread of practical terahertz (THz) systems dedicated to the telecommunication, pharmacy, civil security, or medical markets requires the use of mainstream semiconductor technologies, such as complementary metal-oxide-semiconductor (CMOS) lines. In this paper, we discuss the operation of a CMOS-based free space all-electronic system operating near 250 GHz, exhibiting signal-to-noise ratio (SNR) with 62 dB in the direct detection regime for one Hz equivalent noise bandwidth. It combines the state-of-the-art detector based on CMOS field-effect-transistors (FET) and a harmonic voltage-controlled oscillator (VCO). Three generations of the oscillator circuit are presented, and the performance characterization techniques and their improvement are explained in detail. The manuscript presents different emitter–detector pair operation modalities, including spectroscopy and imaging.

## 1. Introduction

In recent decades, the development and application of technologies operating in the so-called terahertz (THz) electromagnetic frequency band (0.3–10 THz) have attracted considerable attention. The waves at these frequencies are penetrable through many materials, but are non-ionizing; there are many characteristic spectral lines of various materials in the THz range [1]. Various fast and efficient THz wave detecting, generating, and absorbing components and devices are needed to build practical THz systems. However, most existing systems consist of large discrete elements: all-electronic multipliers with sophisticated waveguides [2], continuous-wave telecommunication laser-driven photomixers [3], bulky solid-state pulse laser-driven TDS systems [4], Golay cell, pyroelectric [5] or optoacoustic [6] device, or even cryogenic-cooling bolometers [7] in detectors role.

THz systems’ mass production dedicated to the telecommunication, pharmacy, civil security, or medical markets requires the use of mainstream semiconductor technologies, such as complementary metal-oxide-semiconductor (CMOS) lines. The CMOS technology is up-and-coming due to its readiness for large-scale integration or implementation of power combining techniques using integrated array antennas for higher output power [8]. One of the significant breakthroughs in applying silicon field-effect-transistor (FET) based devices for the THz detection field came in 2009. Then the concept of plasmonic mixing earlier proposed by Dyakonov and Shur [9] allowed efficient detection of radiation frequencies, which often exceed the cut-off frequencies of FETs was demonstrated in a standard CMOS foundry technology [10]. Since then, the understanding of device properties and concomitant performance of CMOS-based detectors was constantly improved, and by now, it is in direct competition with the performance values previously accessible only for mature Schottky diode technology [11,12,13]. The progress in the field of development of silicon circuits for THz frequency range was also achieved at the side of radiation sources reporting radiation powers up to 9 dBm at 250 GHz range [14] and reaching up to 1.4 THz for varactor-based multipliers [15].

Despite significant advances in stand-alone CMOS sources and sensors, attempts to develop THz-based all-CMOS systems have begun late enough. One of the first such systems was demonstrated in 2015 [16]. The 220 GHz all-electronic raster-scan imaging system was based on a differential Colpitts oscillator with an on-chip dipole antenna and a MOSFET-based THz detector with a planar patch antenna. The system was manufactured using 90-nm and 150-nm Si CMOS processes and achieved an SNR of 20 dB. The next year, Statnikov et al. [17] presented a multicolor imaging system with a chipset implemented in a 250-nm SiGe HBT BiCMOS process. The system could operate simultaneously at six harmonics, being multiple numbers of 165 GHz, and achieved an SNR of 115 dB for the 330 and 495 GHz bands, however, in a heterodyne detection regime. The same scientist group continued developing all-CMOS terahertz imaging applications and created a 500 GHz computed tomography based on a commercially available 130-nm SiGe-BiCMOS technology [18]. The used asymmetric zero-bias NMOS detector [19] was needed with chopping to avoid a flicker noise. Thus, the whole system demonstrated 62 dB voltage SNR at 5 kHz modulation frequency compared to a 38 dB SNR in a continuous-wave acquisition mode. Recently, Jain et al. [20] implemented a similar CMOS process to build up a 420 GHz source system-on-a-chip and applied it for computational imaging with a single-pixel camera and spatial modulation of the THz radiation. A 60 dB voltage dynamic range was achieved for one 64-pixel image with a 100 ms acquisition time.

All mentioned systems have consisted of free-space THz emission propagation elements: a planar antenna integrated into the same source and detector chips, a Si substrate lens, discrete optics for beam collimation and focusing such as PTFE lenses [18,20], elliptical mirrors [17], or off-axis parabolic mirrors [16]. Coupling a high-frequency active element to an antenna radiating into free space is a high-challenging task and represents a so-called hybrid electronic class. It should be noted that on-chip hybrid solutions in THz electronics are not fully explored and still have great potential for application, especially in THz spectroscopy, high-resolution imaging, or telecommunications [21,22]. Furthermore, the physical phenomena that gain in importance at nanoscale dimensions allow exploiting different regimes of operation of transistors such as nanoplasma enabled picosecond switches [23] or the emission from collapsing field domains [24] allowing them to reach high power levels. Nonetheless, one important reason to keep the focus on obviously slower and less powerful silicon devices is the readiness of the technology to form a large-scale, collaborative, and flexible clusters, which have the potential to sometimes become even more powerful than many other terahertz technologies [25].

In this paper, we present a new free space all-electronic system operated at 250 GHz. It demonstrates a 62 dB power SNR with 1 Hz equivalent noise bandwidth (equal to 123 dB voltage SNR) in the direct detection regime. It combines the state-of-the-art CMOS detector with a harmonic voltage-controlled oscillator (VCO) and is implemented in 65-nm and 90-nm CMOS processes. The techniques of performance improvement and harmonics filtering are explained in detail. The practical applications for imaging are also demonstrated. Furthermore, despite the fact that here we concentrate on the lower edge of the THz frequency range, a comparative performance can be expected up to 1.4 THz as this is a current limit for silicon-based sources [15] as the detectors of this kind are already proven to be effective up to 9 THz [16].

The paper is divided into four parts. In Section 2, the detector characterization details are presented. Three different methods for the estimation of detector performance parameters are discussed. In Section 3, the source architecture is introduced. The VCOs’ modeling and simulations results, experimental characterization details are also presented. Section 4 is dedicated to emitter–detector pairs performance characterization. The information on the application aspects and the demonstration of the THz imaging example are given. The manuscript is concluded by Section 5.

## 2. FET-Based Resonant Detector

In our study, we employed a resonant-antenna-coupled FET quasi-optical detector (TeraFET) with a substrate lens. It was fabricated using the commercial 90 nm silicon CMOS process of Taiwan Semiconductor Manufacturing Company (TSMC, Taiwan, China) accessible through the Europractice platform. The detector has been designed to have a maximum of response at 250 GHz and was reported previously [26,27]. Here we will present the extended analysis of detectors’ performance.

### 2.1. Characterization Methods

**Figure 1:**
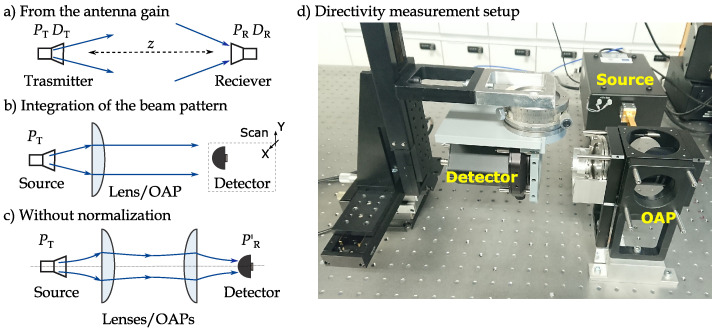
(**a**–**c**) The methods for the detector performance parameters characterization. (**d**) The detector directivity measurement setup. OAP, off-axis parabolic mirror.

In current literature, at least six different methods are reported to specify the performance of antenna-coupled THz detectors [28]. Different possible application scenarios can explain the main reason behind such variety. Therefore, for the sake of comparison with previously published works on similar kinds of detectors and, especially, their application as emitter–detector pairs, we employed three of them: two methods are based on the incident power normalization using the detector’s effective area, and one-without any normalization procedures. The principal schematics of each setup are depicted in Figure 1. Shortly, all methods differ in the estimation of the power delivered into the detector circuit $P_{R}$, which continues to be used in the equation of the voltage responsivity—the proportionality quantity between the THz detector’s voltage response $V_{\mathrm{signal}}$ and the THz power:

(1) $${ℜ}_{V}=\frac{V_{\mathrm{signal}}}{P_{R}}.$$

*Method I. Estimation using the antenna gain*. This method is probably, the most accepted in the field of devices with microwave antennas. It presumes the knowing of the intensity $I_{\mathrm{THz}}$ of radiation at the detector plane, and the antenna gain $G_{R}$. Then, we can calculate the antenna effective area $A_{\mathrm{eff},I}$ using simple equation $A_{\mathrm{eff},I}=G_{R}\lambda_{0}^{2}/4\pi$, where $\lambda_{0}$ is the free-space radiation wavelength ([29], pp. 1–10). The incident power is considered as:

(2) $$P_{R}=A_{\mathrm{eff},I}\cdot I_{\mathrm{THz}}=\frac{G_{R}\lambda_{0}^{2}}{4\pi }\cdot I_{\mathrm{THz}}.$$

If the THz beam has the Gaussian form, the radiation intensity $I_{\mathrm{THz}}$ in the center can be estimated from the total power $P_{T}$ and a beam waist radius *w* at the detector plane as $I_{\mathrm{THz}}=2P_{T}/\left(\pi w^{2}\right)$. The *w* can be gathered from the beam XY profile measurements. In this case, $w=w_{\mathrm{FWHM}}/\sqrt{2\ln2}$, were $w_{\mathrm{FWHM}}$ is the full width at half maximum. The alternative way for the estimation of the *w*—to apply Gaussian beam propagation equation $w=w_{0}\cdot \sqrt{1+{\left(z/z_{R}\right)}^{2}}$, where *z* is the distance between the source and the detector, $z_{R}=\pi w_{0}^{2}/\lambda_{0}$—the Rayleigh range, and $w_{0}$—the beam waist at the source antenna plane.

The gain $G_{R}$ includes the antenna efficiency η—the factor that describes what part of power is delivered into the rectifying device’s circuit. However, both parameters’ experimental estimation is not trivial, especially when the antenna for the THz frequency range is coupled with quasi-optical elements. Thus, the EM simulated gain values are used for the responsivity calculation.

If antenna gain is not determined, the maximal directivity approach for the effective area estimation should be applied ([30], p. 92). The gain and directivity are linked to each other as $G_{R}=\eta D_{R}$. Thus, the equation $A_{\mathrm{eff},I}=D_{R}\lambda_{0}^{2}/4\pi$ does not include the loss of power due to antenna inefficiency; however, it allows estimating power, which is incident on the antenna’s effective area. Furthermore, the antenna’s directivity is much easier to experimentally assess than gain, thus being used reasonably to compare different detectors’ optical characteristics.

*Method II. Estimation using the integration of the beam pattern*. The previous method is applicable when the THz radiation intensity in the center is known. We also can estimate the responsivity by measuring power density patterns and integrate them (Figure 1b):

(3) $${ℜ}_{V}=\frac{1}{A_{\mathrm{eff},\mathrm{II}}\cdot P_{T}}\int V_{\mathrm{signal}}\cdot dX\cdot dY.$$

here, dX and dY are resolutions of the beam scan, accordingly, in X and Y direction. The antenna effective area can be calculated using previously described formulas (using $G_{R}$, or $D_{R}$). The alternative option is applying a physical area of the detector. In this paper, we specified the $A_{\mathrm{eff},\mathrm{II}}$ as the Si lens’s aperture $A_{\mathrm{eff},\mathrm{II}}=\pi \cdot R_{\mathrm{lens}}^{2}$, where $R_{\mathrm{lens}}$ is a radius of quasi-optical element.

It is important to mention that the profile integration technique gives more accurate results for collimated THz beams. The divergent ones have a non-planar wavefront; thus, the usual planar XY scan shows a slightly overestimated detector responsivity. Therefore, we used one off-axis parabolic mirror for the beam collimation in the part of detector characterization experiments. This optical element is depicted as the equivalent lens in Figure 1b.

*Method III. Estimation without any normalization*. A wide range of applications utilizing point-to-point emitter–detector configuration requires the detector’s maximal sensitivity regarding the total power available in the directed (collinear) THz radiation beam. Although any previously mentioned methods can adequately characterize a detector, the focus might be given to system performance. Therefore, the performance of optimized devices can be presented by referring it to the total available power, i.e., without estimation of the effective detector’s area. The later case serves well for direct comparison of the optical performance with calibrated commercially available devices such as bolometers, pyroelectric sensors, Golay cells, or even quasi-optically coupled Schottky diode detectors.

We estimated the power $P_{R}^{\prime }$ at the plane of a detector with a calibrated optoacoustic meter. The simplified diagram of setup is depicted in Figure 1c. The detector was placed in the focal point of the second, focusing off-axis parabolic mirror. The RMS response $V_{\mathrm{signal}}$ was recorded with a spectrum analyzer Stanford Research SR785. The responsivity ${ℜ}_{V}$ is simply calculated as the ratio of two measured quantities ${ℜ}_{V}=V_{\mathrm{signal}}/P_{R}^{\prime }$.

### 2.2. Detector Characterization Results

We used three detector characterization methods described in Section 2.1. In all cases, the all-electronic multiplier-based THz source fabricated by Virginia Diodes Inc. was employed. It was tuned to 252 GHz and radiated the 69 µm power into the free-space through a diagonal, 46 mm-long, WR-2.8 band horn antenna (also fabricated by VDI). The main antenna parameters provided by the manufacturer: a 24 dB gain and 1.9 mm beam waist radius $w_{0}$. The total power $P_{T}$ is measured by the Thomas Keating Ltd. calibrated optoacoustic detector with an aperture of 42 × 68 mm${}^{2}$ placed at the 5 cm distance from the source device.

**Figure 2:**
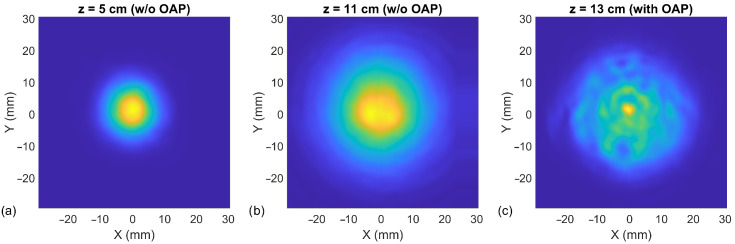
VDI source beam profile in X and Y direction at different distance *z* between the source and the detector at 252 GHz. Two different measurement setups were used: without additional optics ((**a**,**b**) panels) and with one off-axis parabolic mirror ((**c**) panel). The last one corresponds to Figure 1b.

Figure 2a,b shows the spatial THz beam profiles recorded with our resonant FET-based detector by employing an XY motorized scanning stage and taken at 5 cm and 11 cm distances. The resolution step of the scan was 0.5 × 0.5 mm${}^{2}$. As expected, the radiated beam had a vertical linear polarization with an approximately Gaussian intensity distribution perpendicular to the propagation direction. According to the measurement, the radiation beam’s diameter *w* at half-power (−3 dB) points is 11.5 mm at a 5 cm distance from the source, and 20.0 mm—at 11 cm. The estimated waist using antenna parameters provided by VDI is, accordingly, 10.5 mm and 22.4 mm. The difference between experimental and theoretical values can be explained with the beam wavefront curvature at the shortest distance. Because we scanned beam profiles in the XY plane, it resulted in a wider diameter.

**Figure 3:**
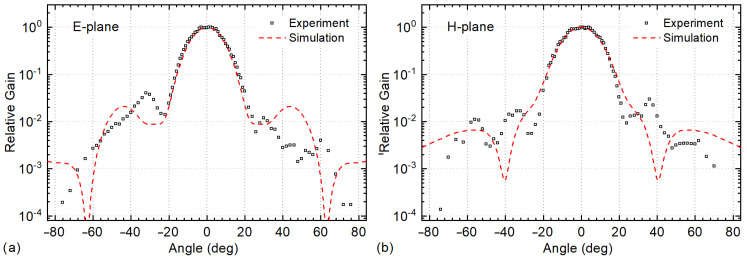
Dependency of the THz response of the resonant detector on (**a**) E-plane and (**b**) H-plane tilt angles at 252 GHz. The simulated response is displayed with the dashed line.

The responsivity was calculated using the measured power density profile at 5 cm distance and Equations (1) and (2) (Method I). We extracted the $G_{R}=18.2$ dB from the EM simulation using the finite element method with adaptive mesh and CST Studio Suite software. The antenna model consisted of the detector planar metal antenna and the 4 mm diameter hyper-hemispherical Si lens coupled from the substrate side. This lens was used in the majority of our beam profile XY scanning experiments. The validity of EM simulation results was checked experimentally—measuring the detector directivity. The directivity was determined by recording the rectified voltage as a function of the device’s tilt angles in the E- and H-plane. The measured radiation patterns are shown in Figure 3. The modeled patterns are shown as a dashed line in the same figure. The measured and modeled main lobe FWHM angles are in good agreement: accordingly, 19.5° vs. 17.9° in the E-plane, and 21.0° vs. 20.2° in the H-plane.

The calculated effective antenna area is 7.5 mm${}^{2}$, which gives 786 *V*/*W* of responsivity and 8.8 pW/$\sqrt{Hz}$ of NEP.

We also estimated the detector sensitivity using the integration of the measured power density pattern (Method II). We employed the scanning of the collimated beam profile. For this experiment, an additional 4"-f off-axis parabolic mirror was used. Furthermore, an 8.9 dB attenuator was applied for the suppression of backward reflections. The result of the scan at 13.25 cm distance from the mirror is depicted in Figure 2c. After applying (3) and the 4 mm Si lens’s aperture as the effective area of the antenna, we got the 427 *V*/*W* responsivity and 16.2 pW/$\sqrt{Hz}$ NEP. If the divergent beam profiles, measured without any optics, are used, the integration technique gives worse sensitivity parameters: accordingly, 417 *V*/*W* for responsivity and 16.6 pW/$\sqrt{Hz}$ for NEP.

There are literature reports in which the characteristics of devices utilize known (either simulated or measured) directivity, i.e., the so-called gain or directivity de-embedded device characteristics for the omnidirectional antenna case (i.e., $A_{\mathrm{omni}}=\lambda_{0}^{2}/4\pi$) [31,32,33]. There is a relation between the area defined in this way and the circularly-shaped diffraction-limited spot. Applying this method for (3), we yield $A_{\mathrm{omni}}=0.11$ mm${}^{2}$, 47.6 kV/W for the responsivity, and 0.15 pW/$\sqrt{Hz}$ for NEP. There are no similar literature values; thus, our THz detector can be treated as the state-of-the-art among FET-based devices.

The detector performance estimation using Method III gave the most conservative values: 408 *V*/*W* for the responsivity and 22 pW/$\sqrt{Hz}$ for the NEP at 252 GHz. We employed the setup shown in Figure 1c, which consisted of two 4”-f off-axis parabolic mirrors and a 12 mm diameter Si lens. The modulation frequency was 1733 Hz. No normalization was used for the estimation procedure. The detector was biased at the point with the minimum noise-equivalent power, i.e., 475 mV gate voltage.

The evaluation of performance based on different methods for effective area estimation of the previously described CMOS detector is presented in Table 1. One can note that the technique with gain de-embedding leads to about two-fold higher reports of the detector’s performance compared with the methods without de-embedding. Due to the selection of focusing mirrors, the optical performance (presented in the last line) is lower than any cross-sectional performance. Nevertheless, another conclusion can be drawn regarding the meaning of 0.15 pW/$\sqrt{\mathrm{Hz}}$, which would result as the NEP for the normalization for the omnidirectional antenna case. As it was not efficiently de-embedded and was much lower than values from applying other methods, it has no direct relevance to either the electrical or optical performance of the devices, besides being embedded again with the directivity value.

We used the last NEP value from Table 1 for the system’s SNR estimation because it best reflects applications, such as raster-scan imaging, spectroscopy, and other systems exploiting point-to-point configurations.

## 3. Colpitts Circuit-Based THz Source

### 3.1. Modeling and Simulations

**Figure 4:**
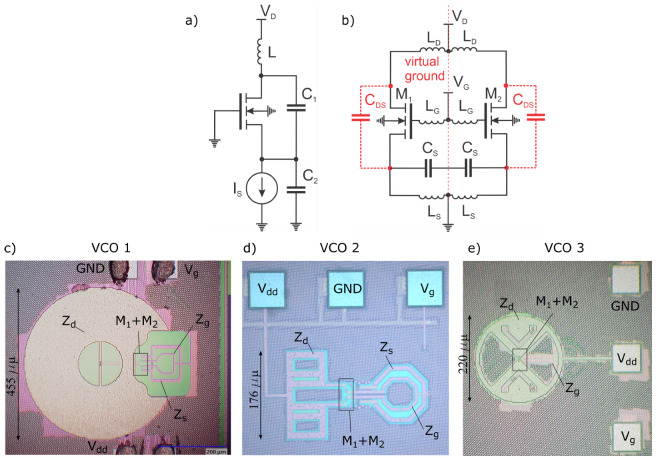
(**a**) The schematic of the basic concept of Colpitts oscillator. (**b**) The proposed VCO is based on the differential configuration of the Colpitts oscillator. Die micrographs of the first VCO ((**c**), area of core 465 µm × 455 µm), the second VCO ((**d**), 284 µm × 176 µm) and the third VCO ((**e**), 305 µm × 220 µm).

The THz source is based on a concept of Colpitts oscillator, which is depicted in Figure 4a. The main oscillation mechanism is based on the common gate transistor amplifier with the positive feedback between source and drain terminals via the capacitor C${}_{1}$. The persistent oscillations occur at the resonance frequency $f_{\mathrm{osc}}=1/\left(2\pi \sqrt{LC_{1}C_{2}/\left(C_{1}+C_{2}\right)}\right)$ with the condition that the voltage gain at this frequency ≥ 4. Figure 4b illustrates a Colpitts oscillator implementation in a differential configuration. The device core is now based on two field-effect transistors $M_{1}$ and $M_{2}$. The feedback capacitor C${}_{1}$ can be physically omitted since the transistor possess substantial drain-to-source intrinsic capacitance C${}_{\mathrm{DS}}$. The capacitor C${}_{2}$ can be replaced by a single capacitor with a capacitance value of $C_{s}/2$ (due to a series connection of two capacitors $C_{s}$). The inductor $L_{s}$ is required for setting DC ground potential. Although for circuit-level simulations, this inductor can be introduced as an RF choke, in practical implementations, an LC-tank with the resonance frequency lower than $f_{\mathrm{osc}}$ gets naturally formed. Furthermore, the replacement of a current source presented in Figure 4a by a fixed gate bias voltage requires introducing an additional inductor $L_{G}$ pair. The existence of inductor $L_{G}$ can result in a possibility to realize a modified Colpitts oscillator circuit [34] consisting just from $L_{G}$, $C_{s}$ and the intrinsic capacitance C${}_{\mathrm{DS}}$. However, if the substantially large inductor $L_{D}$ is connected to the drain terminal, the effective inductance *L* gets replaced by the sum of $L_{D}+L_{G}$. A detailed description of work principals of the first reported differential Colpitts oscillators in a silicon CMOS process for the THz frequency range can be found in [35].

Our circuits were implemented using a 65-nm CMOS process provided by TSMC. The technology offers a stack with nine metal/insulator layers plus top metal layers for passive elements and a p-doped Si substrate with a polysilicon layer for active ones. Figure 4c–e presents three die micrographs with different implemented VCOs (further marked by the VCO1, VCO2, and VCO3). All implementations have been designed to oscillate near the 84 GHz fundamental frequency with the optimized third harmonic emission at about 250 GHz. For all designs, the $L_{G}$ was chosen to be a single loop inductor, and the $C_{S}$ is the capacitor having interdigitated metal-oxide-metal structure. The transistors’ drains are coupled with different implementations of inductors. In all oscillators, we used 14-finger gates, with the length of an individual finger being 60 nm and the width of 2 µm (total width 28 µm). The foundry provided the model’s simulation and revealed that such a transistor should possess a so-called maximum oscillation frequency $f_{\max}$ of 165 GHz for the drain voltage of 1.2 V.

**Figure 5:**
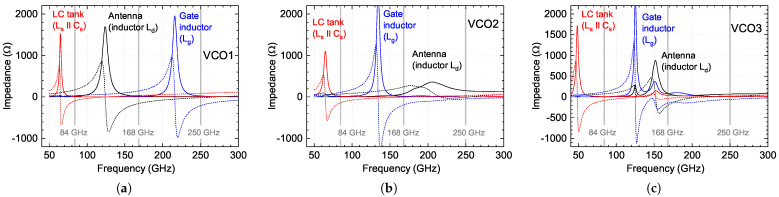
Simulated impedances of VCO core’s elements (inductors, the capacitor, and the antenna) for three proposed device implementations: (**a**)—for VCO1, (**b**)—for VCO2, and (**c**)—for VCO3. The solid lines—a real part, the dashed lines—an imaginary part of the impedance. The vertical gray lines correspond to fundamental (84 GHz), second (168 GHz), and third (250 GHz) oscillation harmonics.

The design presented in Figure 4c employed a resonant, slot-type antenna similar to that as implemented earlier in the text-described detector. It was modified by adding parallel transmission lines to result in 327 pH at 84 GHz and about 7.3 Ω real impedance at 250 GHz for the radiation of generated power at the third harmonics into free-space. Two other designs presented in Figure 4d,e employed elements for $L_{D}$ with lower inductance values at 84 GHz, i.e., 132 pH and 140 pH, yet the careful selection of other circuit elements for all designs ensured oscillation at the nearly identical fundamental frequency as well as its third harmonic. The simulated complex impedance values of all important elements are presented in Table 2 and Figure 5. For these simulations, we employed a Keysigth ADS electromagnetic simulator based on the Method of Moments solver. Since the radiation out-coupling has been intended to be realized from the substrate side by employing hyper-hemispheric substrate lenses similar to those used for detectors, the substrate was simulated as semi-infinite space.

**Figure 6:**
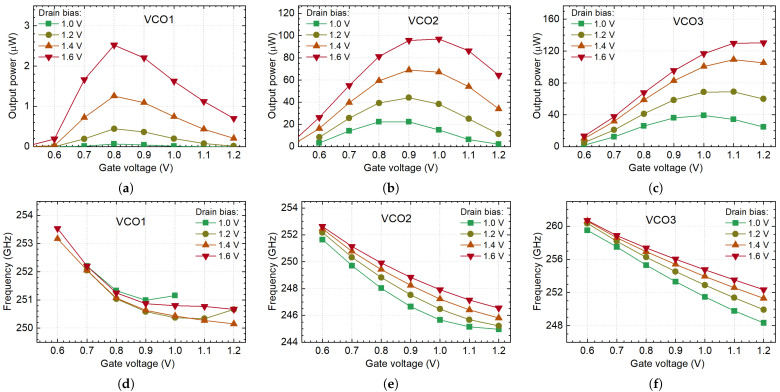
Simulations of third-harmonic output power ((**a**–**c**) panels) and the frequency ((**d**–**f**) panels) as a gate bias $V_{g}$ function for different drain voltages $V_{d}$. The power is estimated including antenna radiation efficiency.

The functionality of the oscillator circuit was simulated using the same Keysigth ADS suite using a harmonic balance solver—a frequency-domain analysis technique. Figure 6 shows the main simulation results—anticipated radiated power and frequency at third harmonics. In the calculations of power, we also included simulated antenna radiation efficiency, which, in our case, was 0.46 for VCO1, 0.6 for VCO2, and 0.56 for VCO3. We employed the finite element method with an adaptive mesh using the CST Studio Suite software to model the antenna structures with a coupled 4 mm diameter Si lens and evaluate the electromagnetic field’s far-field distributions.

The VCO1 model predicts, comparably with the state-of-the-art values (see an overview of [31] and references therein), low output power at the gate bias of 0.85 V: 1.2 µW at 1.4 V of drain bias, and 2.4 µW at 1.6 V. Much better values are predicted for designs VCO2 and VCO3, i.e., 64 µW and 71 µW at 1.4 V, respectively. We attribute the main reason for the predicted low emitted power of VCO1 to comparably lower radiation impedance compared to VCO2 and VCO3, which showed nearly identical circuit performance, differing mainly just in the efficiency of the radiating element $L_{D}$ (antenna).

The predicted frequency at the 3rd harmonics as a function of gate voltage is presented in Figure 6d–f. The simulations show that the emission frequency can be tuned by changing the gate and drain voltages in the range between 250 GHz and 253.5 GHz (corresponds to 1.4% tuning range) for VCO1, and from 243 GHz to 253 GHz (4% tuning range) for VCO2 and the widest 5.2% tuning range (from 248 GHz to 261 GHz) for VCO3.

### 3.2. Experimental Characterization Setup

**Figure 7:**
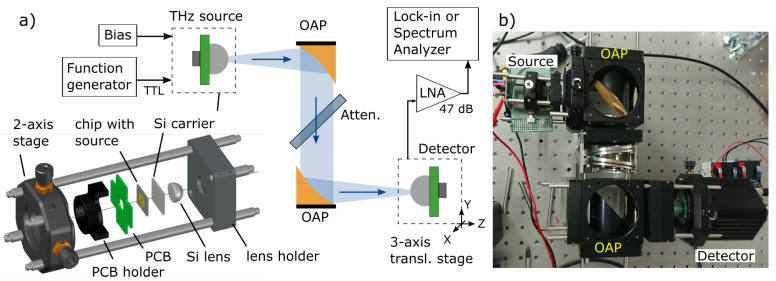
(**a**) Experimental setup for the characterization of the source. Bottom left: the drawing of the source module as used in all experiments. The dashed line represents the setup’s optical axis with the THz beam emitted by the THz source and propagating from the left through the substrate lens to free-space. The chip is not to scale. (**b**) The photo of the characterization system. OAP, off-axis parabolic mirror; PCB, printed circuit board; LNA, low-noise amplifier; Attn., attenuator.

The experimental characterizations and the validation of simulated circuits have been performed in the experimental setup as shown in Figure 7a. The source module was packaged using Thorlabs 30 mm cage system components and own-manufactured adapters. The VCO circuit chip was glued to 500 µm Si wafer (a carrier) as an intermediate medium for the hyper-hemispherical Si lens with a 12 mm diameter and a 6.8 mm height. This wafer is also glued to a printed circuit board (PCB). The PCB holder was used to connect the chip with the Si carrier to the X-Y translation stage. The last one lets us change the VCO position regarding fixed Si lens, and as a result of this to tune THz emission direction. We used the hyper-hemispherical Si lens to reduce a THz beam’s divergence and minimize losses due to internal reflections on silicon-air boundaries [36]. The free-space propagating Gaussian beam is collimated and then focused with two off-axis parabolic mirrors (OAP). The selection of focal length and diameter depends on the transmission distance. We used mirrors with a 4 inch reflected focal length and a 2-inch diameter for all characterization experiments and the imaging application where the distance exceeds 1 m. The 18 dB attenuator is designated to suppress the feedback to VCO, which originates from the reflections from the THz detector.

For our characterizations, we employed three different THz detectors. The calibrated power meter manufactured by Thomas Keating Ltd. was used to evaluate the total integral emitted power consisted of all harmonics and thermal radiation. For lower radiation power levels investigation, the Tydex’s Golay cell was employed. This device had much lower NEP than the first one; however, it exhibited a smaller entrance window. Therefore, it attenuated frequencies below 150 GHz, nevertheless also registering the first harmonic of VCO under test. The FET-based resonant THz detector was designed for optimal performance at 250 GHz and measured only the third harmonic. The 3-axis translation stage was used to center detectors regarding the THz beam or scan the intensity distribution in XY or YZ planes.

**Figure 8:**
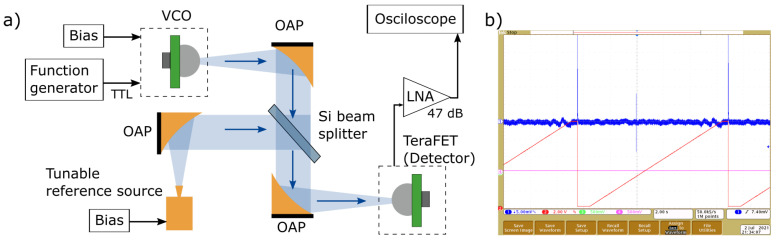
(**a**) Experimental setup for the measurement of the source frequency. (**b**) The photo of the typically recorded oscillogram. The red line - the voltage bias sweep of the reference source, the blue line—the detector’s response. The spike at the center indicates the beat frequency. Two spurious signals appear at the beginning and the end of the sweep. OAP, off-axis parabolic mirror; LNA, low-noise amplifier; VCO, voltage-controlled oscillator.

For the measurement of VCO frequency, we employed the heterodyne detection scheme (see Figure 8a). As a second reference source, we used an all-electronic THz multiplier fabricated by RPG Radiometer Physics GmbH. A 500 µm-thick Si beamsplitter and three off-axis parabolic mirrors with 4-inch effective focal length were used to combine, collimate and focus two THz beams onto our resonant FET-based detector. The reference source was swept in a frequency range from 240 up to 270 GHz. The response of TeraFET was recorded with an oscilloscope; then, the beat frequency was extracted manually from an oscillogram. The typical measurement result is showed in Figure 8b.

### 3.3. Emission Maps

**Figure 9:**
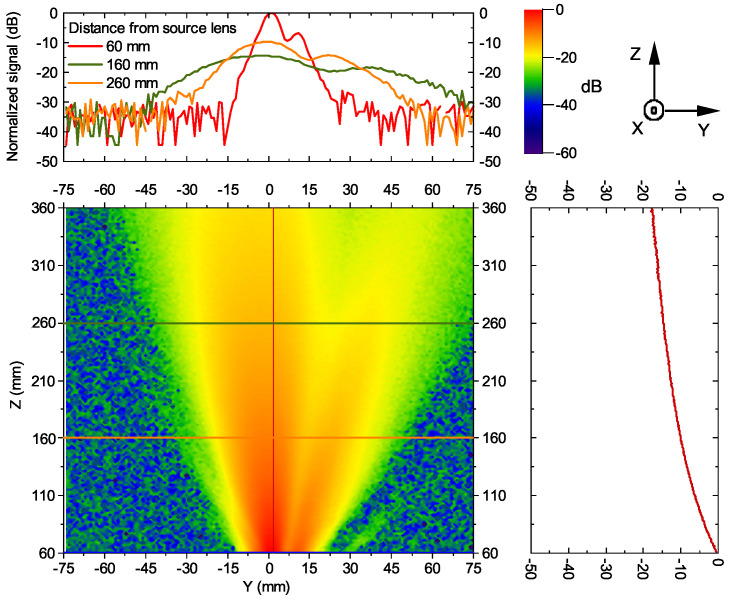
Beam profile of VCO1 in the Y and Z direction, measured with a resonant detector (the setup similar to depicted in Figure 1b, but does not contain the mirror/lens). The data is normalized to the maximum signal. Top: horizontal cross-sections at different distances from the source lens. Right: vertical cross-section at the middle of the THz beam.

We started to investigate our devices from the measurements of a far-field beam profile using the characterization Method II (see Figure 1b). The FET-based detector with 4 mm diameter and 2.1 mm height Si lens was used for these purposes. Any other optics except Si lenses were not used for this experiment. The results of measured intensity distributions for VCO1 in two directions are presented in Figure 9. The device was biased with 1.6 V drain voltage and 0.82 V gate voltage. The main and side lobes of emission can be distinguished, although the power of the second beam is about ten times smaller than the first one. We associated it with the fundamental harmonic emitted from a different place of the chip. The used resonant detector has about 1166× smaller responsivity at 84 GHz than near the 252 GHz frequency. However, the simulated power of 1st harmonic signal is 414 µW at used VCO1 bias values compared with 2.4 µW of 3rd (experimental data to support this estimation will be addressed in the following sections). As a result of this, both harmonics can induce signals with experimentally observed amplitudes. To investigate this issue in more detail, we recorded maps of detected signal strength by raster scanning the VCO chip position on the substrate lens, kept at a fixed position.

**Figure 10:**
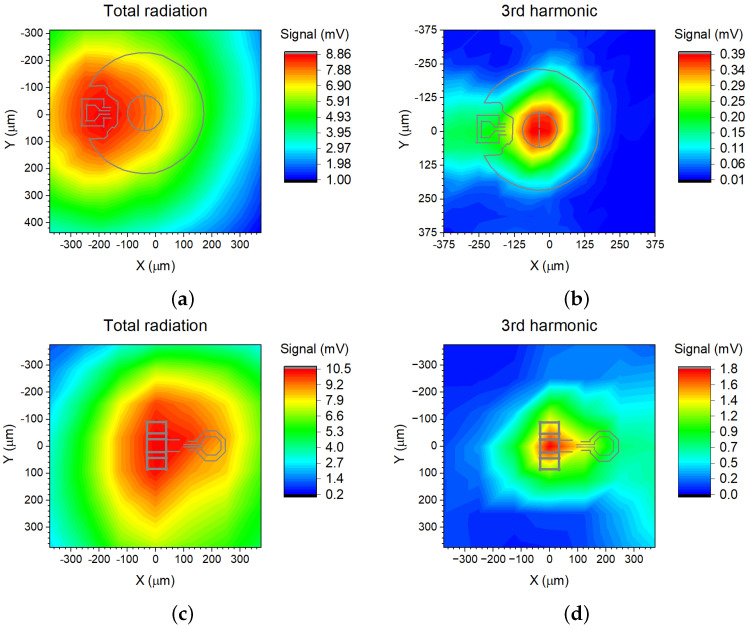
Radiation dependency on the Si lens position on the VCO1 (**a**,**b**) and the VCO2 crystal (**c**,**d**). The total radiation was measured using the Golay cell and the point-to-point setup (see Figure 1c). The 3rd harmonic signal was separated using the same Golay cell and WR-03 (220–325 GHz) conical horn antenna. The depicted VCO structures are simplified versions of ones presented in Figure 4c,d.

The spatial distribution of detected THz radiation signal when the detector and VCO substrate lens are kept at fixed positions and only the VCO crystal position is raster-scanned are presented in Figure 10. We used the Golay cell to measure the total integrated power combining both the fundamental and third harmonic emission. The results for VCO1 are presented in Figure 10a. Since the 1st harmonic signal is much larger than the third one, this XY scan mainly shows the fundamental beam profile. The principal VCO structure is also presented as the reference. To map only the 3rd harmonic radiation, we employed a WR-03 (220–325 GHz) standard waveguide conical horn antenna from Virginia Diodes Inc. as a high-pass filter. It was placed directly in front of the Golay cell. The measured XY profile is depicted in Figure 10b. It is seen that for VCO1, the 254 GHz radiation is emitted from the middle part of the antenna. However, our results indicate that the emission center of the 84 GHz signal is near the center of the gate and source inductors in VCO1.

We also measured a 3rd harmonic radiation distribution in the VCO1 crystal with the FET-based THz detector coupled to the 12 mm Si lens. It exhibited a similar XY profile like that shown in Figure 10b with the maximum signal near the source’s antenna center. These additional results are not presented here; however, it is worth noting that its interpretation is more complicated than ones measured with the Golay cell and the horn antenna due to peculiarities of the setup consisting of two quasi optic elements—12 mm Si lenses.

The difference in radiation center locations of separate harmonic signals on the VCO1 crystal has one positive side effect. It is easier to separate the third harmonic from the fundamental one employing simple Si lens positioning techniques such as a justification procedure with a compact 2-axis translation stage or placing and gluing the optical element using a commercially available pick-and-place station. For comparison, we also present the XY scans of the VCO2 chip (see Figure 10c,d). Despite the more efficient suppression of the amplitude of the fundamental oscillation in this design, both harmonics are radiated from the same place—the center of the drain’s antenna. Therefore, more sophisticated filtering techniques should be involved when the practical application would require spectral purity. For example, the resonant TeraFET detectors as employed by us can be efficiently utilized as a high-pass filter for separation of 3rd harmonic. However, we would like to address the current challenge that evaluating the radiated power with commercially available calibrated broadband detectors is more complicated.

### 3.4. The Emitted Power

**Figure 11:**
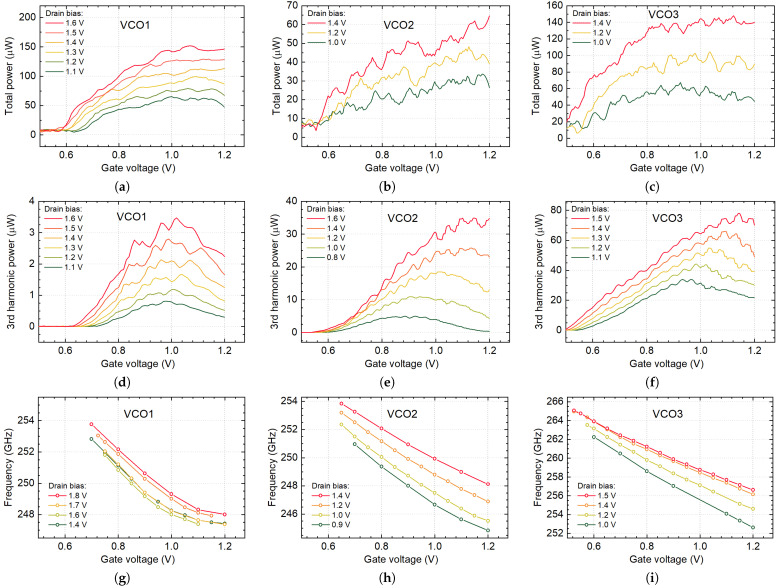
The proposed VCOs’ measured total power (**a**–**c**), 3rd harmonic power (**d**–**f**), and frequency (**g**–**i**) dependency on the gate $V_{g}$ and drain $V_{d}$ biases.

The total and third harmonic radiation power of implemented devices as a function of the gate and drain voltages are presented in Figure 11a–f. The total power, which consisted of up all harmonics and the thermal radiation, was measured with the calibrated optoacoustic detector manufactured by Thomas Keating Ltd. when the 3rd harmonic signal—with our resonant TeraFET. The on-off keying of $V_{d}$ with a function generator provided a 26 Hz and 1733 Hz, respectively, and electrical modulation was used.

As expected from the simulation results, the large amount of the emitted radiation power can be attributed to the fundamental oscillations in the VCO1. The power of the 3rd harmonic signal is 1.3 µW (−28.4 dBm) in comparison with 90 µW (−10.5 dBm) of the total power at 1.4 V of the drain voltage and 0.85 V of the gate bias (see Figure 11a,d). The maximum radiated third harmonic power of 4.7 µW, or −23.3 dBm has been measured, though the VCO1 bias condition ($V_{d}=$ 1.8 V and $V_{g}=$ 1.03 V) is not practical due to excess heating and increased FET breakdown probability. Thus, we used sources for the imaging application at a working point lower than maximum. The circuit consumed DC power from 6.6 mW at $V_{d}=$ 1.4 V and $V_{g}=$ 0.82 V to 29 mW at $V_{d}=$ 1.8 V and $V_{g}=$ 1.2 V.

The VCO2 exhibited a higher third harmonic emission in comparison with VCO1 (Figure 11b,e). Moreover, VCO2 excels better at first harmonic suppression—15.4 µW (−18.1 dBm; 3rd harmonic) vs. 45 µW (−13.5 dBm; total power) at 1.4 V of the drain voltage and 0.85 V of the gate bias. The maximum radiated third harmonic power was 35 µW (−14.6 dBm) at $V_{d}=$ 1.6 V and $V_{g}=$ 1.1 V. The VCO3 out-performed both predecessors in emission power and tunability measurements (Figure 11c,f). The 3rd harmonic power was 43 µW (−13.7 dBm), the total power—139 µW (−8.6 dBm) at 1.4 V the drain voltage and 0.85 V at the gate bias. The maximum radiated power of 78 µW (−11.1 dBm) has been measured, at the VCO3 bias condition $V_{d}=$ 1.5 V and $V_{g}=$ 1.15 V.

It should be pointed out that the presented VCO radiation power values are estimated at the end of the 30 cm length optical system on the detector side. Only attenuation loss of 18 dB in the case of third harmonic measurements is considered in the calculation.

The measured frequency at the 3rd harmonics as a function of gate voltage for all VCOs is presented in Figure 11g–i. The emission frequency can be tuned in the range between 247.5 GHz and 254 GHz (corresponds to 2.6% tuning range) for VCO1, from 245 GHz to 254 GHz (4% tuning range) for VCO2, and the widest 5.2% tuning range (from 252 GHz to 265 GHz) for VCO3. The measured tuning range represents the bandwidth of the emitter within which we were able to observe stable oscillations.

The simulated and measured power and frequency of VCO2 and VCO3 are in good agreement. The simulation of VCO1 shows oscillation amplitudes reach their maximum at $V_{g}=$ 0.8 V, with a decrease in the gate voltage (compare Figure 6a and Figure 11a). However, our measurements show more complicated behavior of VCO1. The manufactured chip exhibited the maximum of 3rd harmonic power shifted toward bigger $V_{g}$ values (≈1 V); moreover, it has two maximums similar in amplitude. The total power of all three oscillators does not decline with the drain voltage increase when $V_{d}>$ 1.4 V. We associate this behavior with the chip heating, which increases in high $V_{g}$ and $V_{d}$ region.

Figure 11d–f excels one interesting and useful feature—periodic oscillations in curves. They can be employed to calculate the tunability of VCO. The oscillations of measured power are caused by the formation of standing waves—the interference between two beams traveling in forward and backward directions. The VCO and the detector form the resonating system (see Figure 7a). Despite the usage of the attenuator, which suppresses backward reflections, the formation of standing waves still occurs in the measurement system and could be observed even at low bias conditions.

When the gate voltage of VCO is changed, the frequency of propagating THz beam is also changed. It is easy to calculate the difference in frequencies δf of two adjacent standing waves (*n* and n+1; n=1,2,3,…), which is equal to $c/(2\cdot l_{0})$, where $l_{0}$ is the distance between the emitter and the detector, *c*—the speed of light. In our setup, $l_{0}$ was 0.3 m, what correspond to δf = 500 MHz. As can be calculated from Figure 11e, the VCO2 frequency changed by 500 MHz when we raise $V_{g}$ by 0.05 V when $V_{d}$ = 1.6 V. For lower drain voltage, the period of curve oscillation is dropping to 0.044 V at $V_{d}$ = 1.4 V, and 0.039 V at $V_{d}$ = 1.2 V. These values are in very good agreement with direct frequency measurements results, presented in Figure 11h. The tunability of VCO is wider at lower drain voltage. Thus, the period of peaks in power measurements curves is smaller.

## 4. Performance of Emitter-Detector Systems

### 4.1. Signal-to-Noise Ratio

The signal-to-noise ratio or SNR is traditionally defined as a ratio of the power of a signal $P_{\mathrm{signal}}$ to the power of background noise $P_{\mathrm{noise}}$. For the systems relevant to this manuscript, the term power of a signal applies to the power of the incident THz radiation $P_{\mathrm{THz}}$, which relates with either voltage or current response produced at the detector’s output. The proportionality is called responsivity and is marked using German type Fraktur letter *ℜ* with an index *V* or *I* for voltage or current signals correspondingly, i.e., voltage response $V_{\det}={ℜ}_{V}\cdot P_{\mathrm{THz}}$. The background noise is represented by the electrical fluctuations at the detector output, which can originate from the statistics of incident photon numbers or be determined by the electrical fluctuations produced by the detector’s circuitry. As long as the later is the case, then the electrical noise voltage $V_{N}$ determines the minimal detectable power of incident radiation, i.e., $P_{\mathrm{noise}}=V_{N}/{ℜ}_{V}$. When the noise voltage is additionally normalized to the equivalent noise bandwidth of one hertz, the $P_{\mathrm{noise}}$ obtains a well known definition of noise equivalent power (NEP), i.e., $\mathrm{NEP}=V_{N}/\left({ℜ}_{V}\cdot \sqrt{∆f}\right)$ with ∆f being the bandwidth. The SNR can also be expressed in logarithmic decibel scale:

(4) $$\mathrm{SNR}\left(\mathrm{dB}\right)=10\cdot \log_{10}\left(\frac{P_{\mathrm{signal}}}{P_{\mathrm{noise}}}\right)=10\cdot \log_{10}\left(\frac{V_{\det}}{V_{N}}\right).$$

It is worth noting that based on the definition of responsivity, the detector’s SNR gets directly related with the ratio between detected voltage $V_{\det}$ and $V_{N}$. The later relation can produce confusion by a natural desire to apply an SNR equation for amplitudes:

(5) $${\mathrm{SNR}}_{V}\left(\mathrm{dB}\right)=20\cdot \log_{10}\left(\frac{V_{\mathrm{signal}}}{V_{\mathrm{noise}}}\right),$$

which is applicable when the $V_{\mathrm{signal}}$ and $V_{\mathrm{noise}}$ are, respectfully, the signal and noise amplitudes. This is the case for THz amplitude sensing techniques such as heterodyne detection of subharmonic mixing but should not be applied for direct detectors. Sometimes, the device’s parameter defined by (5) is called a voltage signal-to-noise-ratio, and one defined by (4)—a power SNR.

**Figure 12:**
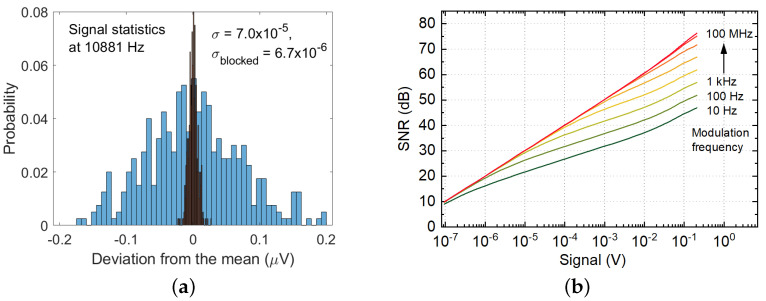
(**a**) Statistics of the detector output signal in two cases: in normal measurement mode (blue wider bars) and with a blocked source (black, narrower bars). (**b**) The modeled dependency of the detector SNR on the signal strength and modulation frequency at a carrier frequency of 250 GHz.

For the source-detector system as an opto-pair characterization, we employed the VCO3 and the 12 mm diameter hyper-hemispherical Si lens coupled to both active elements to reduce a THz beam’s divergence and minimize losses. The free-space propagating Gaussian beam is collimated and then focused with two 4${}^{\prime \prime }$-f off-axis parabolic mirrors (OAP). The distance between the source and the detector was 35 cm. Figure 12a presents statistics of the detector output signal with the unblocked and blocked source. The maximum achievable signal-to-noise ratio for the system with VCO3 is 61.7 dB for 1 Hz ENBW (equivalent noise bandwidth). For a 3 ms integration time (ENBW = 83.3 Hz), the estimated power SNR is 52 dB. We used the “power decibels” Equation (4) to estimate system SNR, which is more conservative than “voltage decibels”. If we use conversion to dB units strategy as described in [17,20], the maximum SNR would be twice bigger—123.4 dB; thus, our optopair is setting the state-of-the-art level.

At high SNR levels, the rectified signal also introduces a substantial increase in detector noise. It is demonstrated in Figure 12a when the signal of 0.6 V at detector output at 10,881 Hz modulation frequency results in about a ten times increase of standard variation compared with blocked conditions. The dependence of expected SNR as a function of the detected signal for different modulation frequencies is shown in Figure 12b. It clearly demonstrates the appearance of an excess 1/f type noise, which corner frequency increases with the increase of the incident power or committed detected signal. Above the corner frequency, the SNR and the precision of amplitude determination become equal.

### 4.2. Application to Imaging System

**Figure 13:**
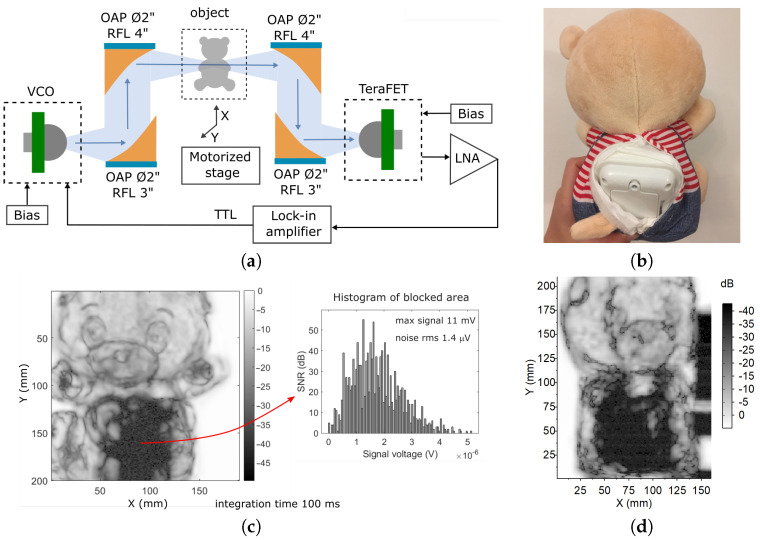
(**a**) Terahertz raster imaging system. (**b**) The photo of the sample. (**c**) THz image of the sample recorded in the setup with VCO1 and the Si lens of 4 mm diameter. Inset: a histogram of recorded values in a blocked region allowing to estimate the noise voltage. (**d**) The THz image of the same sample was recorded with the setup with more powerful VCO3 and the Si lens of 12 mm diameter. RFL, reflected focal length; OAP, off-axis parabolic mirror; LNA, low-noise amplifier.

With a view towards future low-cost, practical applications, we applied our all-CMOS opto-pair to terahertz imaging. The simple raster scanning techniques were employed. Our 4${}^{\prime \prime }$-f raster imaging system includes one of described Si CMOS VCO’s, the resonant Si FET-based detector, and four Ø2${}^{\prime \prime }$ off-axis parabolic mirrors. The THz radiation is focused on an object and collected after by using two mirrors with reflected focal length (RLF) of 4${}^{\prime \prime }$; mirrors for VCO and detector have RLF 3${}^{\prime \prime }$ as shown in Figure 13a. The VCO’s radiation is modulated by a 1 kHz TTL signal from the same lock-in amplifier that registers the signal from TeraFET. It permits us to achieve a dynamic range in transmission measurements of more than 40 dB in power for a time constant of 100 ms. An object for raster imaging is mounted onto a motorized linear translation XY-stage. This setup corresponds to the device characterization Method III.

The two-dimensional raster-scan images of the teddy bear are shown in Figure 13c. The images comprise 4 k pixels covering different focal plane areas in the range of 90 mm for head and 150 mm for length, a thinner part as the hand has 30 mm thickens. It was composed of non-woven fabric and is covered by soft velour. The toy has a sensor at extremities and a voice module inside the body that is shown in Figure 13b. We used Ø12 mm hyper-hemispherical lens in the source module. This lens is well coupled to a Gaussian-beam system, and couples to a converging beam [37]. At the same time, the hyper-hemispherical lens could effectively increase the gain of the TeraFET integrated antenna by $n^{2}$ as theoretical maximum, where *n* is the refraction index of the lens [38].

In the case of Figure 13c, VCO1’s radiation is focused to TeraFET by the hyper-hemispherical lens with Ø4 mm. For Figure 13d, we use the same Ø12 mm lens as for VCO and try to achieve the maximum possible signal after the object; also, a more powerful emitter VCO3 is used. These improvements allow an increased maximum of free space signal over 20 dB, where 13 dB is power increasing, and 7 dB is the effect of the lens. However, the increase in SNR does not directly contribute to the improvement of image quality, as it can be seen in Figure 13d. First of all, the presented image’s dynamics are limited by a 40 dB dynamical range of lock-in amplifier. Furthermore, the blurring of finer features originated from more effective radiation collection compared to a stronger effect of diffraction, which operates as a visual edge enhancement technique for a system used in Figure 13c. Note that the image with a better visual appearance has been obtained with the weakest source VCO1, which emits only a fraction of power compared to VCO3. Therefore, the design of a specific application-oriented imaging system is a complex task that requires both: optimization of the source-detector pair efficiency and the accurate integration of these elements into a complete quasi-optical imaging system.

## 5. Conclusions

In summary, this paper presents the compact all-electronic THz system that is entirely based on silicon CMOS technology. Three different designs of the voltage-controlled oscillator in the role of the THz source are presented. The simulated and measured performance characteristics, such as emission power and frequency, are demonstrated. Furthermore, the spatial distribution of radiation of separate harmonic signals on the VCO crystal is presented, and the application of the difference in radiation center locations is discussed. One FET-based device coupled with the resonant planar antenna is employed as a direct power sensor. Three different methods are demonstrated to specify the performance of antenna-coupled THz detectors, and different possible application scenarios of each characterization are explained. The estimation of the performance of the whole opto-pair system is presented, and signal-to-noise ratio calculation aspects are discussed. The system features 252 GHz operational frequency and up to 62 dB power SNR with 1 Hz equivalent noise bandwidth. Finally, the practical application of the CMOS quasi-optical THz source-detector pair for two-dimensional imaging is also demonstrated; optimizing the source-detector pair efficiency and the accurate integration of elements into a complete quasi-optical imaging system are explained.

## Figures and Tables

**Table 1:** Comparison of responsivity and NEP of the FET-based detector at 252 GHz, considering different methods calculating the effective area and/or the impinging power.

Method	$A_{\mathrm{eff}}$	Maximum ${ℜ}_{V}$	Minimum NEP
	(mm${}^{2}$)	(V/W)	(pW/$\sqrt{\mathrm{Hz}}$)
I. From the antenna gain	7.5	786	8.8
IIa. Integration of the beam pattern	12.6	417	16.6
IIb. Integration of the beam pattern with directivity de-embedded	0.11	47.6 k	0.15
III. Without any normalisation (optical performance)	N/A${}^{*}$	408	22.0

${}^{*}$ Not applicable.

**Table 2:** Comparison of simulated impedances of VCOs at fundamental and third harmonic frequencies.

Circuit Element	At 84 GHz			At 250 GHz		
	**VCO1**	**VCO2**	**VCO3**	**VCO1**	**VCO2**	**VCO3**
Source LC ($L_{s}\left|\right|C_{s}$)	10.0−i94.8	19.4−i146.4	8.4−i86.5	5.7+i87.0	4.6+i35.0	9.0−i19.5
Drain inductor ($L_{d}$)	17.6+i172.9	3.6+i70.0	3.4+i74.7	7.3−i17.1	150.0−i6.0	69.2+i20.9
Gate inductor ($L_{g}$)	3.9+i67.4	11.6+i107.4	8.4+i122.3	44.8−i419.3	4.9−i49.9	9.5−i58.6

## Data Availability

The data that support the findings of this study are available from the corresponding authors (K.I. and A.L.) upon reasonable request.

## Sample Availability

Detectors and sources can be available for testing after contacting the authors.

## Abbreviations

The following abbreviations are used in this manuscript:

BiCMOS	Bipolar CMOS
CMOS	Complementary metal–oxide–semiconductor
ENBW	Equivalent noise bandwidth
FET	Field–effect transistor
FWHM	Full width at half maximum
HBT	Heterojunction bipolar transistor
LNA	Low-noise amplifier
MOSFET	Metal–oxide–semiconductor field-effect transistor
NEP	Noise equivalent power
NMOS	N–type metal–oxide–semiconductor
OAP	Off-axis parabolic mirror
PCB	Printed circuit board
PTFE	Polytetrafluoroethylene
RLF	Reflected focal length
SNR	Signal–to–noise ratio
TDS	Time-domain spectroscopy
TeraFET	Field–effect transistor–based THz detector
THz	Terahertz
VCO	Voltage–controlled oscillator
PTFE	Polytetrafluoroethylene
